# Effect of Salt Intake on Plasma and Urinary Uric Acid Levels in Chinese Adults: An Interventional Trial

**DOI:** 10.1038/s41598-018-20048-2

**Published:** 2018-01-23

**Authors:** Yang Wang, Chao Chu, Ke-Ke Wang, Jia-Wen Hu, Yu Yan, Yong-Bo Lv, Yu-Meng Cao, Wen-Ling Zheng, Xi-Long Dang, Jing-Tao Xu, Wei Chen, Zu-Yi Yuan, Jian-Jun Mu

**Affiliations:** 1 Department of Cardiology, First Affiliated Hospital of Medical School, Xi’an Jiaotong University, Xi’an, China; 2 Key Laboratory of Molecular Cardiology of Shaanxi Province, Xi’an, China; 3 Department of Clinical Laboratory, First Affiliated Hospital of Medical School, Xi’an Jiaotong University, Xi’an, China

## Abstract

Uric acid (UA) has been proposed as an important risk factor for cardiovascular and renal morbidity. We conducted an interventional trial to assess effects of altered salt intake on plasma and urine UA levels and the relationship between UA levels and salt sensitivity in humans. Ninety subjects (18–65 years old) were sequentially maintained on a normal diet for 3 days at baseline, a low-salt diet for 7 days (3.0 g/day, NaCl), and a high-salt diet for an additional 7 days (18.0 g/day of NaCl). Plasma UA levels significantly increased from baseline to low-salt diet and decreased from low-salt to high-salt diet. By contrast, daily urinary levels of UA significantly decreased from baseline to low-salt diet and increased from low-salt to high-salt diet. The 24 h urinary sodium excretions showed inverse correlation with plasma UA and positive correlation with urinary UA excretions. Additionally, salt-sensitive subjects presented significantly higher plasma UA changes in comparison to salt-resistant subjects, and a negative correlation was observed between degree of salt sensitivity and plasma UA difference. The present study indicates that variations in dietary salt intake affect plasma and urine UA levels, and plasma UA may be involved in pathophysiological process of salt sensitivity.

## Introduction

Excess dietary salt induces adverse cardiovascular and renal effects according to epidemiological, interventional, and experimental studies^1–3^. Hypertension prevention studies have proven that moderate salt reduction promotes a 25% long-term reduction of risk for cardiovascular events; thus, salt intake is related to cardiovascular functions^4^. Several mechanisms, including endothelial dysfunction, oxidative stress, inflammation, insulin resistance, and a neurogenically mediated increase in peripheral resistance, contribute to the harmful effects of dietary salt^5–8^.

Serum uric acid (UA) is an important factor in the causal pathway for gout^9^. Blood UA has also been implicated as a potential risk factor and/or mediator of cardiovascular disease outcomes and mortality in a large number of observational studies^10,11^. Although most clinical trials targeting serum UA reduction have focused on pharmacological interventions^12,13^, diet has long been identified as an important determinant of circulating UA levels. Recently, a few studies have shown that increased sodium intake significantly lowers serum UA^14,15^. However, no research has studied the relationship between dietary salt intake and UA levels, especially urinary UA excretion.

Salt has been linked to hypertension for many years. Heterogeneous blood pressure (BP) response to changes in dietary salt intake, generally referred to as salt sensitivity, is regarded as an intermediate phenotype of essential hypertension and is observed in both hypertensive patients and normotensive individuals^16^. Salt-sensitive (SS) individuals account for more than 50% of hypertensive patients or normotensive individuals with a positive history of hypertension, whereas individuals with a negative history account for approximately 27%^17^. Previous studies indicated that SS subjects are confronted with severe target organ damage and high morbidity and mortality of cardiovascular disease for normotensive or hypertensive individuals^18–21^. An association of UA and salt sensitivity has been established in experimental rats for two major phases^22,23^. However, data on the link between UA levels and salt sensitivity are lacking in humans.

In this study, we prospectively examined the effects of salt intake on plasma and urine UA in normotensive and mildly hypertensive subjects. We also investigated the correlation between UA levels and salt sensitivity.

## Methods

### Subjects

Subjects with similar dietary habits were screened from 2 villages in Liquan and Lantian Counties, Shaanxi Province, China. Ninety of the 102 subjects were enrolled in this study. Twelve subjects were excluded due to diabetes and kidney disease or unwilling participation. Sample size was calculated based on the frequency of evacuation and standard deviation of the difference as 0.8 between periods^24^. A total sample size of 90 was sufficient to expect a 95% power with a two-sided significance level of 0.05. A brief medical questionnaire was administered. Hypertension was defined as systolic BP ≥ 140 mmHg, diastolic BP ≥ 90 mmHg, or both or as the use of antihypertensive medications in concordance with recommendations from the Seventh Joint National Committee on Prevention, Detection, Evaluation, and Treatment of High Blood Pressure^25^. BP levels can increase after dietary intervention. Therefore, we excluded those with stage 2 hypertension. The other exclusion criteria comprised secondary hypertension; history of clinical cardiovascular disease, chronic liver and kidney disease, or diabetes; use of antihypertensive medication; pregnancy; and high alcohol intake.

Participants completed questionnaires, which gathered demographic information and detailed information on health-related behaviour, diagnoses of cardiovascular and renal disease, medication use, and family history. In addition to BP measurements, participants were assessed for height and weight and provided fasting blood samples and 24 h urine specimens.

The research was approved by the Ethics Committee of the First Affiliated Hospital of Medical School, Xi’an Jiaotong University (Code: 2015–128) according to the Declaration of Helsinki (2008) of the World Medical Association. All participants provided written informed consent. The trial registration number was NCT02915315 (http://www.clinicaltrials.gov), with date of registration 27/09/2016.

### Dietary Intervention

**Figure 1:**
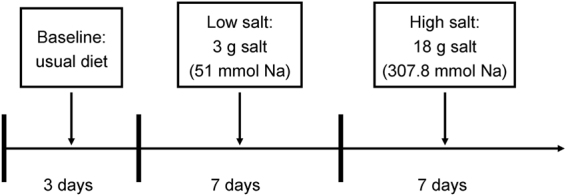
Flow diagram showing the intervention programme.

The chronic salt intake intervention protocol was performed as previously described (Fig. 1)^20,26^. Briefly, during a 3-day baseline period, trained staff collected data during physical examination, including height, weight, waist circumference, and BP measurements. After the 3-day baseline observation, study participants received a 7-day low-salt diet (3 g of sodium chloride or 51.3 mmol sodium per day) followed by a 7-day high-salt diet (18 g of sodium chloride or 307.8 mmol sodium per day). Dietary potassium intake remained unchanged during the two intervention phases. During the baseline investigational period, each subject was provided detailed dietary instructions to avoid table salt, cooking salt, high-sodium foods, and food rich in nitrites/nitrates for the subsequent 14 days. All study foods were cooked without salt, and pre-packaged salt was added to the meals of individual study participants when served by the study staff. To ensure compliance with the intervention programme, participants were required to eat their breakfast, lunch, and dinner in the study kitchen under the supervision of the study staff during the entire intervention period.

### BP Measurement and Definition of Salt Sensitivity

BP levels were measured by three trained staff members using a standard mercury sphygmomanometer. Measurements were performed while subjects were in the sitting position after resting for ≥5 min. For each patient, BP was measured thrice at 1 min intervals during the 3-day baseline observation period and on days 6 and 7 of each of the two 7-day intervention periods. BP observers were blinded to participants’ dietary interventions. Subjects were instructed to avoid alcohol, cigarette smoking, coffee/tea, and exercise for at least 30 min prior to BP measurements. Systolic BP (SBP) and diastolic BP (DBP) were determined through the first and fifth Korotkoff sounds, respectively. Pulse pressure was calculated as SBP − DBP. Mean arterial pressure (MAP) was calculated as DBP+ (1/3× pulse pressure). BP at baseline and during intervention was calculated as the mean of six measurements from two clinical visits during the 3-day baseline observation period and as the mean of measurements on days 6 and 7 of each of the two 7-day intervention periods, respectively. Given the lack of universal consensus on the definition of the salt sensitivity of BP, subjects with a ≥10 mmHg increase in MAP from the low- to high-salt diet were classified as SS and those with a <10 mmHg increase as salt resistant (SR)^20,27^.

### Blood Biochemical Analyses

Blood samples were obtained by peripheral venous puncture, immediately centrifuged at 3,000 × *g* for 10 min, and stored at –80 °C until analysis. Total cholesterol, triglycerides, low-density lipoprotein cholesterol, high-density lipoprotein cholesterol, alanine aminotransferase (ALT), aspartate aminotransferase (AST), serum creatinine, serum Na^+^, K^+^, and Cl^─^, and fasting blood glucose levels were evaluated with an automatic biochemical analyser (model 7600; Hitachi, Ltd., Tokyo, Japan). Plasma UA levels were measured with a Hitachi clinical chemistry analyser using the uricase HMMPS method. Five plasma samples were used to evaluate intra-assay and inter-assay coefficients of variation, which ranged from 2.1% to 4.5% and from 3.2% to 6.4%, respectively, for UA.

### Twenty-four Hour Urinary Electrolytes and Biochemistry

Samples of 24 h urine were collected at baseline and on day 7 of each intervention period. Any urine collection less than 500 mL or with a creatinine excretion lower than the population mean minus two standard deviations was discarded to ensure completeness of collection^28^. Samples were kept frozen at −40 °C until analysis. All urine samples were shipped in ambient packaging with the use of ice boxes to the clinical laboratory at the First Affiliated Hospital of Xi’an Jiaotong University in Xi’an, China. Urinary concentrations of UA, sodium, and potassium were analysed using an automatic biochemical analyser (Hitachi, Ltd., Japan). The 24 h urinary excretions of UA, sodium, and potassium were quantified by multiplying UA, sodium, and potassium concentrations, respectively, by the total 24 h urine volume.

### Statistical Analyses

Continuous data are presented as the mean ± standard error. Categorical data are expressed as frequency with percentage. Given that the levels of ALT, AST, triglycerides, and chloride are not normally distributed, these parameters were expressed as median values and 25% and 75% interquartile ranges. Differences in repeated measures were analysed by repeated-measure analysis of variance. Pearson’s correlation coefficient was used to determine the correlations when residuals were normally distributed, and Spearman’s correlation coefficient was used otherwise. We also performed prespecified subgroup analyses to assess for effect modification by baseline covariates known to influence changes in UA with changes from a low- to high-salt diet; these covariates include age (<60 and ≥ 60 years), sex (male and female), body mass index (BMI) category (<24, 24–28, and ≥ 28 kg/m^2^), baseline plasma UA concentration (<212.5, 212.5–249, 249–293.75, and ≥ 293.75 µmol/L), baseline urinary microalbumin excretion (<6.88 and ≥ 6.88 mg/24 h), and baseline hypertension status. These comparisons were explored to interpret and elucidate the primary results. Statistical analyses were performed in SPSS for Windows, Version 16.0 (SPSS Inc., Chicago, IL, USA). A two-tailed p value of <0.05 was considered statistically significant.

### Data availability

All raw experimental data used in this study are available from the corresponding authors upon request.

## Results

### Profiles of Studied Subjects

All subjects completed intervention trials. Table 1 highlights the demographic and clinical characteristics of study participants. Eighteen subjects (20%) experienced hypertension, and none of them were taking any medication.

**Table 1:** Baseline Demographic and Clinical Characteristics.

Parameters	Values
Age, y	50.5 ± 1.1
Sex (M/F)	32/58
Body mass index, kg/m^2^	24.2 ± 0.3
Waist circumference, cm	85.7 ± 1.0
Smoking (n, %)	23 (25.6)
Hypertension (n, %)	18 (20)
Pulse, bpm	73.4 ± 1.1
Systolic blood pressure, mmHg	116.9 ± 1.7
Diastolic blood pressure, mmHg	75.3 ± 1.0
Mean blood pressure, mmHg	89.2 ± 1.1
ALT, U/L*	22 (19.9∼28.2)
AST, U/L*	19.5 (16.0∼31.1)
Fasting blood glucose, mmol/L	4.58 ± 0.11
Total cholesterol, mmol/L	4.33 ± 0.10
Triglycerides, mmol/L*	1.19 (0.86∼1.56)
LDL-cholesterol, mmol/L	2.42 ± 0.07
HDL-cholesterol, mmol/L	1.33 ± 0.04
Serum creatinine, μmol/L	55.5 ± 0.9
Serum Na^+^, mmol/L	141.2 ± 0.2
Serum K^+^, mmol/L	4.36 ± 0.07
Serum Cl^−^, mmol/L*	104.2 (102.7∼105.4)
Plasma UA, µmol/L	260.5 ± 8.0

Values are means ± SE or percentages; ALT: alanine aminotransferase, AST: Aspartate aminotransferase,

LDL: low-density lipoprotein, HDL: high-density lipoprotein, UA: uric acid. *Expressed as median (25–75%).

### Effects of Salt Intake and Potassium Supplementation on BP and 24 h Urinary Sodium and Potassium Excretion

At baseline, 24 h urinary sodium and potassium excretion averaged 172.1 ± 7.6 mmol/day (approximately 10.1 g of salt per day, 1 g salt = 17.1 mmol Na) and 37.9 ± 2.0 mmol/day (approximately 0.97 g of K per day, 1 mmol = 39 mg K), respectively. These data suggest that the dietary pattern of the Northern Chinese population is characterized by a high sodium intake and an insufficient intake of potassium, agreeing with our previous survey^20,21^. Table 2 presents BP responses to low-salt and high-salt interventions. Overall, BP levels significantly increased with the change from low-salt to high-salt intervention (*P* < 0.05).

**Table 2:** BP Levels (mmHg) and 24-h Urinary Sodium and Potassium Excretions (mmol/d) at Baseline and During Dietary Interventions.

	SBP	DBP	MAP	24 h urinary Na^+^	24 h urinary K^+^
Baseline	116.9 ± 1.7	75.3 ± 1.0	89.2 ± 1.1	172.1 ± 7.6	37.9 ± 2.0
Low-salt diet	112.4 ± 1.3^†^	75.6 ± 0.9	87.9 ± 0.9	91.2 ± 4.0^†^	34.4 ± 1.6
High-salt diet	122.0 ± 1.9*	78.7 ± 0.9*	93.1 ± 1.2*	266.7 ± 7.5*	37.6 ± 1.7

Values are means ± SE. ^†^P < 0.05 vs baseline; ^*^P < 0.05 vs low-salt diet. BP, blood pressure; SBP, systolic blood pressure; DBP, diastolic blood pressure; MAP, mean arterial pressure.

The 24 h sodium and potassium excretions in urine were calculated after each intervention period to ensure compliance of participants. As shown in Table 2, urinary sodium excretion significantly decreased with the change from baseline to the low-salt diet but increased from the low-salt to the high-salt diet (*P* < 0.05). Urinary potassium excretion did not significantly change among different periods. These results confirmed compliance of the subjects with the dietary intervention protocol.

### Effects of Salt Intake on Plasma and Urinary UA Levels

**Figure 2:**
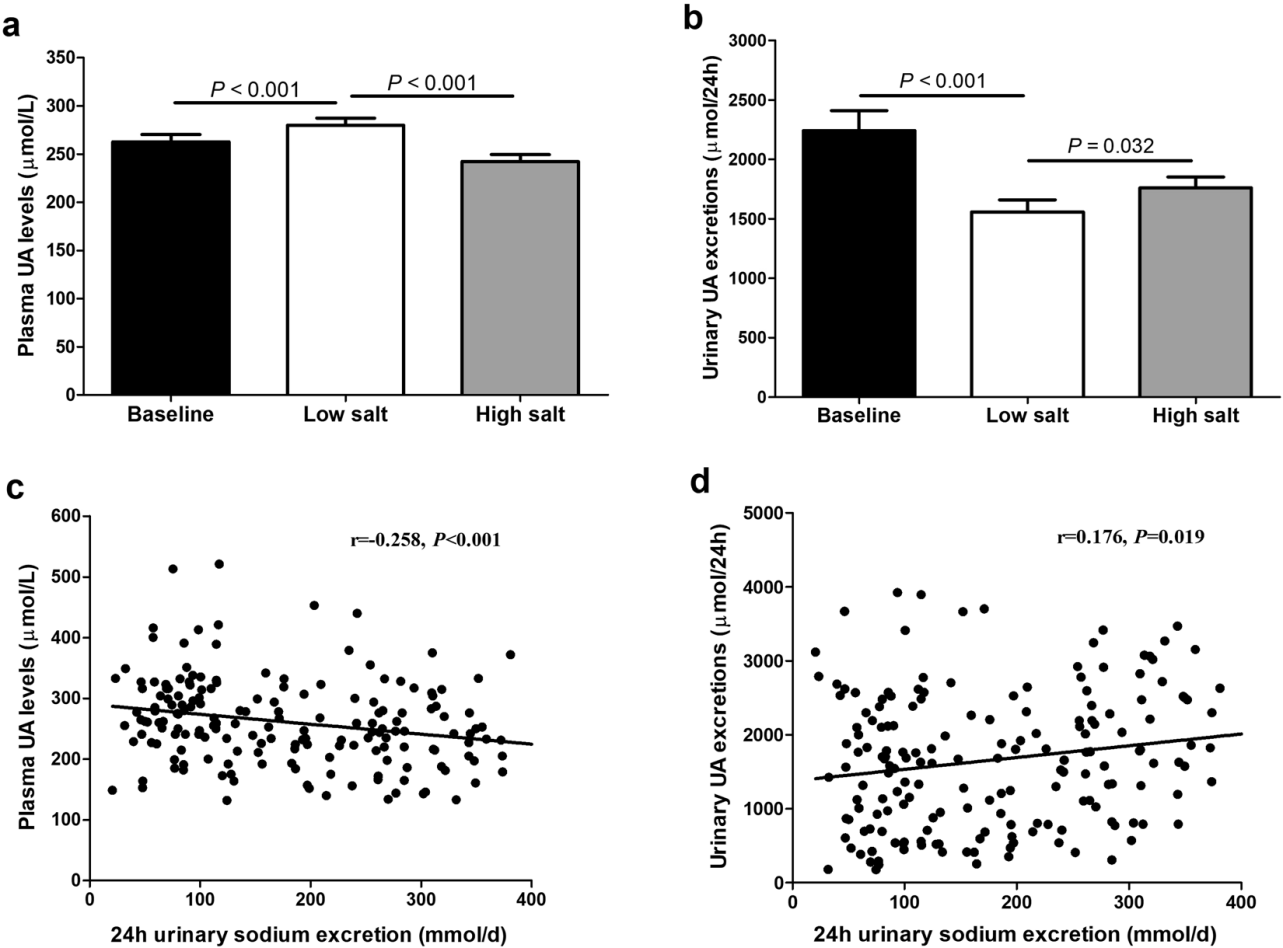
Effects of low-salt and high-salt intake and on plasma (**a**) and urinary (**b**) UA levels in all subjects. Correlations between 24 h urinary sodium excretions and plasma UA levels (**c**), or urinary UA excretions (**d**) in all subjects on a low-salt and on a high-salt.

Plasma UA levels significantly increased with the change from baseline to the low-salt diet (262.7 ± 7.6 vs. 280.0 ± 7.4 µmol/L, *P* < 0.001) and decreased from the low-salt to the high-salt diet (280.0 ± 7.4 vs. 242.6 ± 7.1 µmol/L, *P* < 0.001) (Fig. 2a). By contrast, daily urinary levels of UA significantly decreased with the change from baseline to the low-salt diet (2,242.4 ± 168.3 vs. 1,558.2 ± 101.1 µmol/24 h, *P* < 0.001) and increased from the low-salt to the high-salt regime (1,558.2 ± 101.1 vs. 1,761.9 ± 90.2 µmol/24 h, *P* = 0.032) (Fig. 2b).

Further analyses showed that 24 h urinary sodium excretions were inversely correlated with plasma UA (*r* = −0.258, *P* < 0.001) and positively correlated with urinary UA excretions (*r* = 0.176, *P* = 0.019), during both the low-salt and high-salt diet intervention periods (Fig. 2c and d, respectively).

### Salt Sensitivity and Plasma and Urinary UA Levels

For many years, animal studies have proposed an association between UA and salt sensitivity of BP^29^; however, this area of research remains unclear for humans. Nineteen subjects, who showed increased MAP of ≥ 10 mmHg from the low-salt to high-salt periods, were considered SS. The remaining 71 subjects were considered SR. Table 3 displays the baseline characteristics of the study population according to the SS status. At the baseline, SS subjects were older, associated with high BP levels, and showed high prevalence of hypertension (42.1% vs. 14.1%, *P* = 0.007).

**Table 3:** Characteristics of Salt-Sensitive and Salt-Resistant Subjects.

Parameters	Salt sensitive	Salt resistant	*P*-value
Age, y	57.4 ± 1.3	48.7 ± 1.3	0.001
Sex (M/F)	6/13	26/45	0.683
Body mass index, kg/m^2^	24.7 ± 0.8	24.1 ± 0.4	0.427
Waist circumference, cm	86.0 ± 2.0	85.7 ± 1.1	0.904
Smoking (n, %)	5 (26.3)	18 (25.4)	0.932
Hypertension (n, %)	8 (42.1)	10 (14.1)	0.007
Pulse, bpm	75.4 ± 2.5	72.8 ± 1.2	0.345
Systolic blood pressure, mmHg	114.4 ± 1.8	126.5.9 ± 4.2	0.004
Diastolic blood pressure, mmHg	79.0 ± 2.3	74.3 ± 1.0	0.05
Mean blood pressure, mmHg	94.8 ± 2.7	87.7 ± 1.2	0.009
ALT, U/L	24.2 ± 1.9	25.9 ± 1.2	0.492
AST, U/L	26.7 ± 4.7	27.9 ± 2.2	0.799
Fasting blood glucose, mmol/L	4.74 ± 0.29	4.53 ± 0.17	0.635
Total cholesterol, mmol/L	4.25 ± 0.20	4.35 ± 0.11	0.653
Triglycerides, mmol/L	1.37 ± 0.18	1.36 ± 0.09	0.940
LDL-cholesterol, mmol/L	2.43 ± 0.16	2.42 ± 0.08	0.940
HDL-cholesterol, mmol/L	1.22 ± 0.07	1.36 ± 0.04	0.136
Serum creatinine, μmol/L	56.3 ± 1.8	55.3 ± 1.1	0.666
Serum Na^+^, mmol/L	141.3 ± 0.4	141.1 ± 0.2	0.672
Serum K^+^, mmol/L	4.29 ± 0.13	4.38 ± 0.08	0.619
Serum Cl^−^, mmol/L	104.6 ± 0.5	104.7 ± 0.7	0.973
Plasma UA, µmol/L	233.0 ± 15.3	267.9 ± 9.1	0.075

Values are means ± SE or percentages; ALT: alanine aminotransferase, AST: Aspartate aminotransferase, LDL: low-density lipoprotein, HDL: high-density lipoprotein, UA: uric acid.

**Figure 3:**
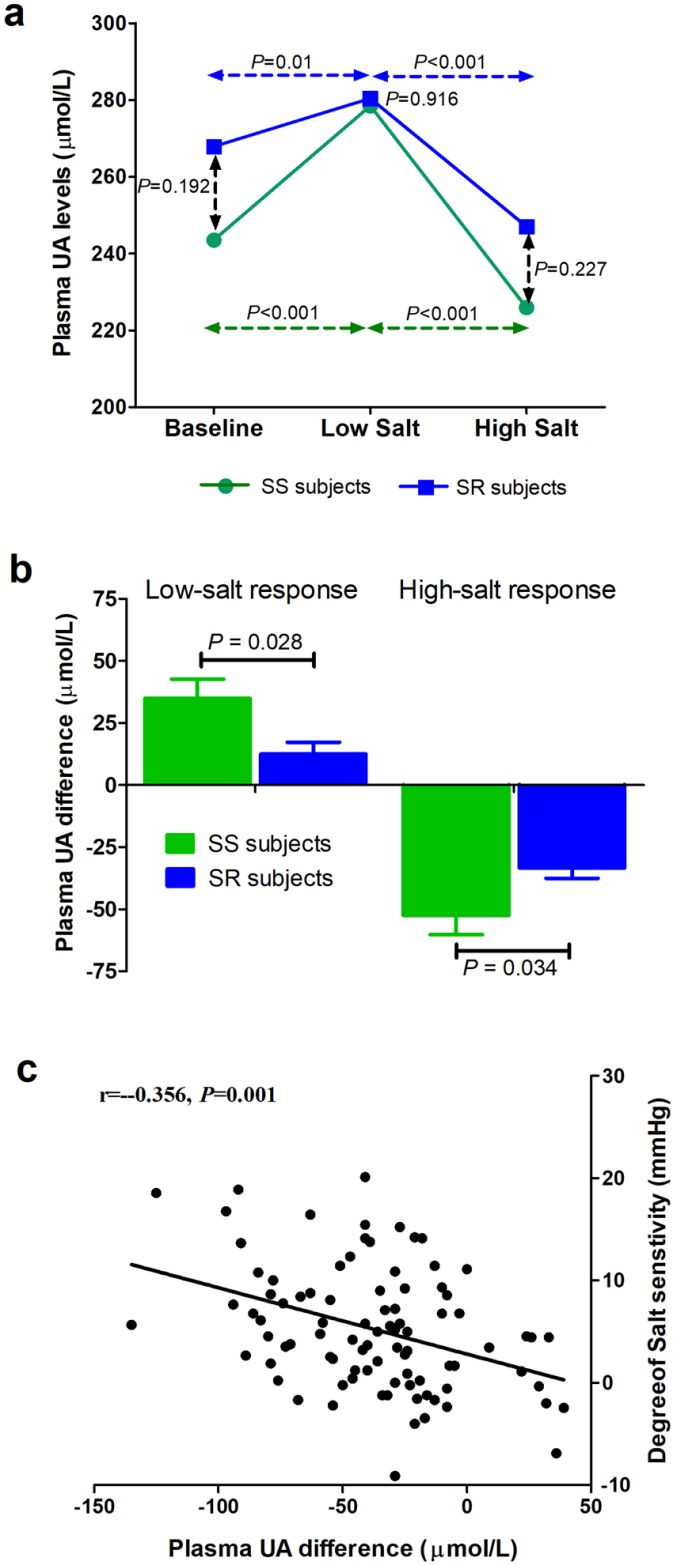
(**a**) Comparison of plasma UA levels at baseline, low-salt and high-salt intake periods in salt-sensitive (SS) and salt-resistant (SR) subjects. Vertical dashed lines represent differences between SS and SR subjects at each of the salt intake levels. Horizontal dashed lines represent differences between salt intake levels (baseline versus low and low versus high). (**b**) Comparison of plasma UA responses to low-salt and high-salt diets in SS and SR subjects. (**c**) Correlation between plasma UA difference and degree of salt sensitivity in all subjects.

As shown in Fig. 3a, plasma UA levels significantly increased with the change from baseline to the low-salt diet and decreased from the low-salt to the high-salt regime in both SS and SR subjects (*P* < 0.05 for all). However, no significant difference was observed in plasma UA between SS and SR subjects during any level of salt intake. To further increase the statistical power of the study, we also compared changes in plasma UA while transitioning from baseline to the low-salt diet and from the low- to high-salt diet. SS subjects were associated with significantly higher plasma UA changes compared with SR subjects (low salt response: 34.9 ± 7.8 vs. 12.5 ± 4.8 µmol/L, *P* = 0.028; high salt response: −52.5 ± 7.7 vs. −33.4 ± 4.1 µmol/L, *P* = 0.034; Fig. 3b). We observed a negative correlation between the degree of salt sensitivity, which is defined as the difference in MAP at the end of the high- and low-salt diet periods, and plasma UA difference (*r* = −0.356, *P* = 0.001; Fig. 3c).

Figure S1a presents the relationship between urinary UA excretions and salt sensitivity. Daily urinary levels of UA significantly decreased with the change from baseline to the low-salt diet and remained stable with the change from the low-salt to the high-salt regime in both SS and SR participants. However, no difference in the changes in urinary UA excretions was observed between the SS and SR groups (P > 0.05 for all; Fig. S1b). Further analyses showed that the degree of salt sensitivity was not related to urinary UA excretion difference (r = –0.075, P = 0.483; Fig. S1c).

### Subgroup analysis

**Figure 4:**
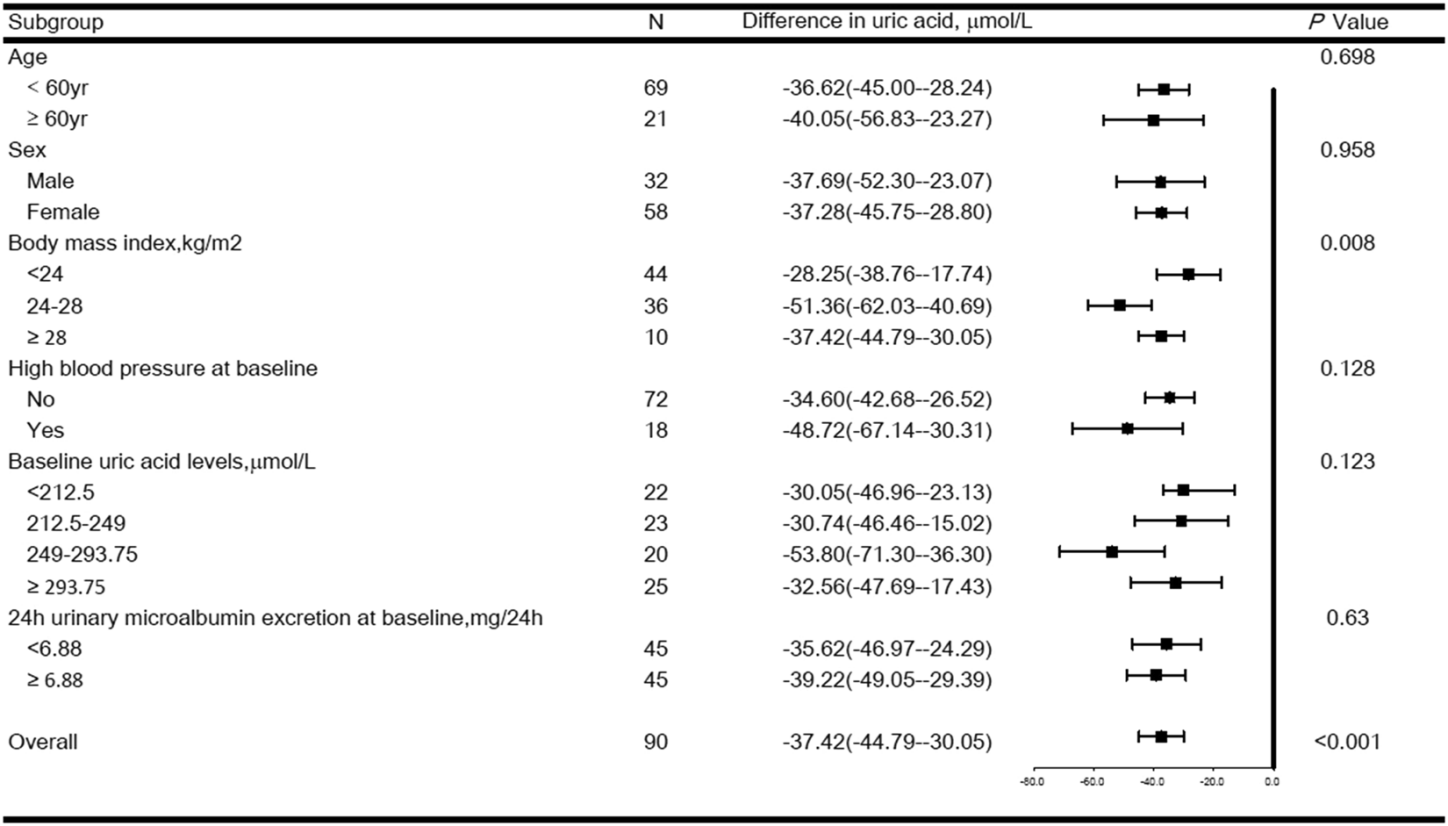
Forest plots of the effects of high (18.0 g/day) versus low (3.0 g/day) salt intake on plasma UA levels in subjects stratified by age, sex, BMI, baseline hypertension status, baseline UA level, and 24-h urinary microalbumin excretions at baseline. Values are the mean (95% confidence interval [95% CI]).

Figure 4 shows the effects of sodium intake on plasma UA, stratified by age, sex, BMI, baseline hypertension status, baseline UA level, and 24 h urinary microalbumin excretions at baseline. Participants with a BMI of 24 kg/m^2^ to < 28 kg/m^2^ demonstrated more significant effects from high versus low salt intake than did participants with a BMI of <24 kg/m^2^ (−51.36 vs. −28.25 µmol/L; *P* = 0.003). The effect of the salt diet on plasma UA was nearly null when baseline plasma UA was either <212.5 or 212.5 µmol/L to <249 µmol/L. However, when baseline plasma UA measured from 249 µmol/L to <293.75 µmol/L, the high-salt diet reduced plasma UA (53.80 µmol/L). This effect was more intensive (53.80 µmol/L) in participants with baseline plasma UA of 249 µmol/L to <293.75 µmol/L than that (32.56 µmol/L) in participants with baseline plasma UA of ≥ 293.75 µmol/L (*P* = 0.044) (Fig. 4). Age, sex, baseline hypertension status, and 24 h urinary microalbumin excretions at baseline caused no changes in the relationship between salt intake and plasma UA.

## Discussion

The results of the present study demonstrate that low salt intake increased plasma UA from the baseline, whereas high salt intake decreased plasma UA levels under a low-salt diet. In addition, an inverse correlation between the 24 h urinary sodium excretion and plasma UA level was demonstrated in these Chinese subjects. These data indicate that variations in dietary salt intake significantly influence plasma UA levels.

Although the relationship between salt intake and hypertension is well-established, its relationship with UA is controversial. One proposed hypothesis states that higher UA levels represent an evolutionary advantage in *Homo sapiens*, allowing them to maintain BP when access to sodium is scarce^22^. This theory was demonstrated in a uricase-deficient rat model showing an increase in BP from hyperuricaemia in the context of a low-sodium diet^23^. Additional rat models have supported this hypothesis, showing that UA causes upregulation and activation of epithelial sodium channels in nephrons^30^. Hou *et al*.^31^ recently reported that high salt intake enhanced associations of blood UA with hypertension and related cardiovascular risk in a cross-sectional study of 1,668 Chinese subjects. However, clinical trials scarcely examine this relationship. One trial performed in 27 men showed that increasing sodium from 20 mEq/day to 200 mEq/day decreased UA levels by 1 mg/dL^32^. In another randomized crossover trial of 103 adults with pre-hypertension or stage I hypertension, 30 days of low versus high sodium intake (60 versus 180 mmol/day) significantly decreased serum UA^14,15^. These observations agree with the results of our study, in which salt loading significantly lowered plasma UA levels, and an inverse association was observed between 24 h urinary sodium and plasma UA. The present study builds upon these previous findings by also evaluating the effects of low salt intake. Low salt resulted in a marked increase in circulating UA levels from baseline, enhancing our knowledge of urate pathophysiology and risk factors for hyperuricaemia.

To our knowledge, this study is the first report in the literature concerning the association between salt intake and urinary UA. We discovered that urinary excretion of UA substantially decreased as the baseline diet changed to a low-salt one but increased from the low-salt to the high-salt regime. The mechanism by which increased sodium intake decreases plasma UA remains to be determined. Increased sodium intake has been noted to cause increased intravascular volume^33^, and this condition can cause haemodilutional effects of sodium on UA. The relationship between sodium intake and UA reduction possibly results from the effects of sodium intake on the glomerular filtration rate and excretion or absorption of urate. Previous physiology studies have shown that reabsorption of sodium and urate accompanies one another at different sites in nephrons^34,35^. Thus, decreased renal reabsorption of sodium from excess sodium intake possibly contributes to decreased urate reabsorption. This hypothesis has been evidenced by our study, showing that urinary UA excretions were markedly increased during high salt intake, which was further reinforced by the observation that urinary UA was positively correlated with urinary sodium excretion. Finally, this relationship may reflect actions of the renin–angiotensin system, as UA is inversely related to vascular resistance and renal blood flow^36,37^. Similarly, angiotensin II has been shown to decrease urate excretion after acute infusion^38,39^. Determining the molecular mechanism and signalling molecules responsible for the effects of salt intake on plasma and urinary UA is of significant interest.

To our knowledge, our study is the first to demonstrate that the plasma UA responses in SS subjects are more significant than those in SR subjects during low- and high-salt periods. A negative correlation was also observed between the degree of salt sensitivity and plasma UA differences in the studied Chinese population. We also noted that urine UA was not associated with salt sensitivity. These data suggest that blood UA may be involved in the pathogenic mechanisms of salt sensitivity. By contrast, ter Maaten *et al*.^40^ showed that serum UA was not associated with salt sensitivity in a small study of 21 healthy volunteers (*r* = 0.31, *P* > 0.05). This relationship has been confirmed by animal studies. In the late 1990s, Johnson and colleagues developed a mild hyperuricaemic model by using oxonic acid, a uricase inhibitor, to increase UA levels. Over the 7 weeks of oxonic acid and low-salt diet, SBP in hyperuricaemic rats increased by 30–40 mmHg, with increased serum UA. Additionally, an increase in BP is linearly related to the rise in UA (*r* = 0.75)^23^. After 7 weeks on a low-salt diet and oxonic acid, when oxonic acid was removed, serum UA and BP fell to normal levels over 3 weeks; however, when hyperuricaemic rats were fed a high-salt diet, they became hypertensive and SS^22^.

In exploratory subgroup analyses, we observed the highest UA reduction from low salt intake to high salt intake in participants with a BMI of 24 kg/m^2^ to <28 kg/m^2^. This subgroup also represents a population that can possibly benefit from a low-salt diet for UA management. A number of studies have shown that higher levels of serum UA are associated with higher BMI, and weight loss may be an effective non-medical strategy for reducing serum UA levels^41–43^. Kuwahara *et al*.^44^ showed that excessive BMI increases during childhood led to young adult serum UA elevation in a linkage study of 298 Japanese children. In the present study, plasma UA levels were not correlated with BMI at baseline (*r* = 0.181, *P* = 0.087; Fig. S2). Different study populations, different sample sizes, and racial differences among these various studies may be causes of discrepant results. Interestingly, participants with a baseline plasma UA of 249 µmol/L to <293.75 µmol/L showed the most significant UA reduction after salt loading. Future studies should investigate mechanisms underlying this phenomenon.

The present study features some limitations. First, the study population was relatively small and restricted to northern Chinese individuals. Therefore, our results will require replication in other cohorts to determine generalizability to other ethnicities and to populations with different backgrounds. Similarly, the study excluded persons with prior cardiovascular disease, advanced kidney disease, and medication-treated diabetes mellitus or hypertension, thus possibly limiting its generalizability. Furthermore, our subgroup analyses are, by nature, exploratory. Differences between the strata of baseline UA levels (high salt intake versus low salt intake) or strata of BMI warrant confirmation in a dedicated trial.

Notwithstanding these limitations, the present study presents a number of important strengths. We employed a randomized trial to examine the effects of dietary interventions on plasma and urinary levels of UA. Further, participation in the dietary interventions was high, and excellent compliance with the study interventions was noted, as assessed by urinary excretions of sodium during each intervention period. Finally, salt sensitivity was analysed as a dichotomous trait (such as the SS and SR groups) and as a continuous variable (degree of salt sensitivity) to increase this study’s power to observe the effects of salt intake on UA within study participants, despite the modest sample size.

For more than 50 years, studies have identified the association between UA and cardiovascular risk. In recent years, increased salt intake has been shown to lower serum UA levels. First, this interventional study shows that low salt intake increased plasma UA and decreased urinary excretion of UA, whereas the high-salt diet decreased plasma UA but increased urine UA. Second, plasma UA and salt sensitivity demonstrated a significant relationship. Most importantly, the significant response of plasma UA after salt intake in SS individuals suggested an association of blood UA with the pathogenesis of salt sensitivity of BP. These results support the evidence obtained in animal studies that mild hyperuricaemia results in salt sensitivity and hypertension in two major phases. Our findings are of public health importance because they provide a basis for potential prevention and a possible therapeutic target for hypertension in the future.

## Electronic supplementary material

Supplementary information 
