# Supplementary material for: Effect of Salt Intake on Plasma and Urinary Uric Acid Levels in Chinese Adults: An Interventional Trial
Source: Sci Rep. 2018 Jan 23;8:1434. doi: 10.1038/s41598-018-20048-2 (PMC5780523; doi:10.1038/s41598-018-20048-2)
Supplement: Supplementary file 1 — Supplementary information [file 41598_2018_20048_MOESM1_ESM.pdf]

Effect of Salt Intake on Plasma and Urinary Uric Acid Levels in Chinese Adults: An Interventional Trial

Yang Wang[1,2†], Chao Chu[1,2†], Ke-Ke Wang[1,2†], Jia-Wen Hu[1,2], Yu Yan[1,2], Yong-Bo Lv[1,2], Yu-Meng Cao[1], Wen-Ling Zheng[1,2], Xi-Long Dang[1], Jing-Tao Xu[1], Wei Chen[3], Zu-Yi Yuan[1,2], Jian-Jun Mu[1,2*]

1. **Department of Cardiology, First Affiliated Hospital of Medical School, Xi'an Jiaotong University, Xi'an, China;**
2. **Key Laboratory of Molecular Cardiology of Shaanxi Province, Xi'an, China;**
3. **Department of Clinical Laboratory, First Affiliated Hospital of Medical School, Xi'an Jiaotong University, Xi'an, China.**

† These authors contributed equally to this work.

*** Correspondence author: Jian-Jun Mu, MD**

Address: Department of Cardiology, First Affiliated Hospital of Medical School, Xi'an Jiaotong University, 277 Yanta West Road, Xi'an, 710061, China.

E-mail: mujjun@163.com

Phone: 86-29-85323804

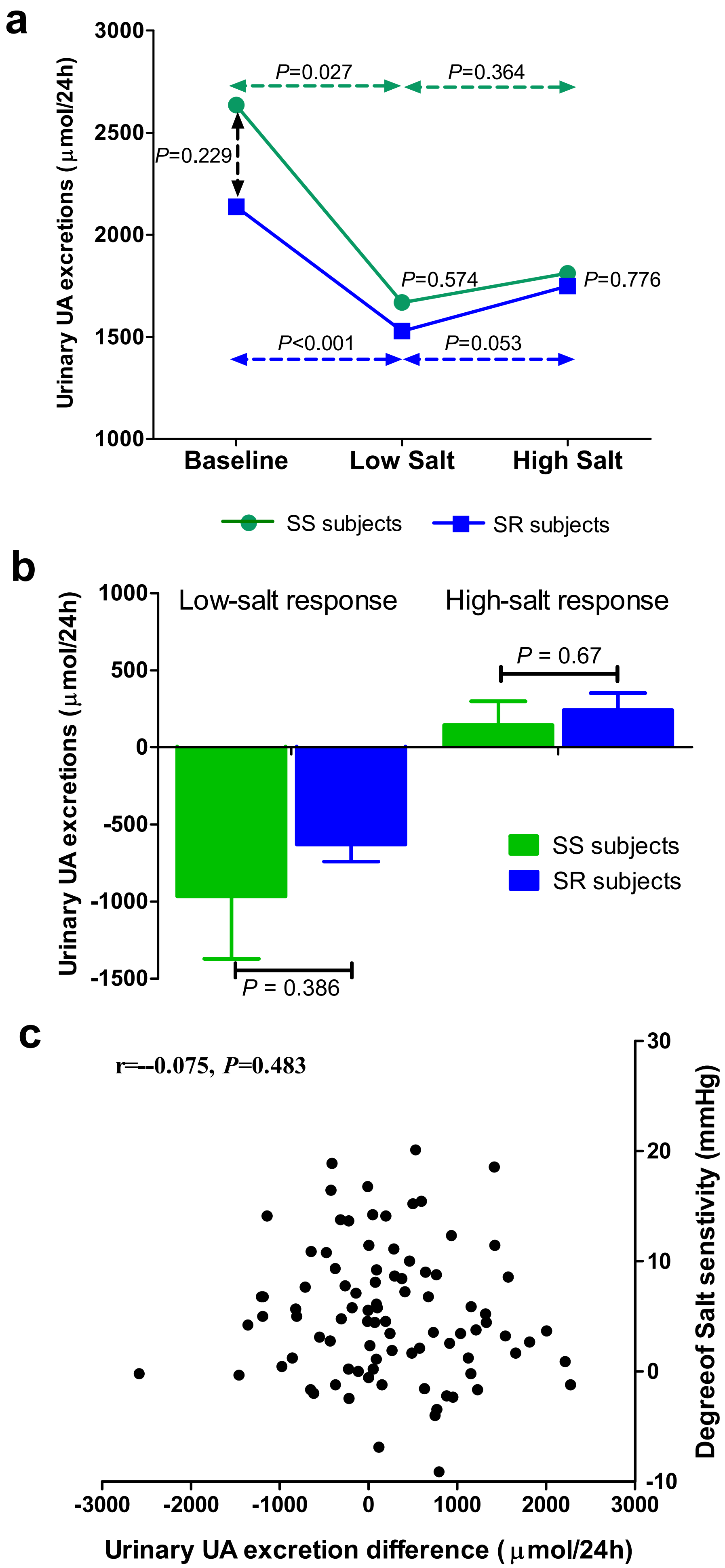

a
3000
2500
2000
1500
1000
Urinary UA excretions (μmol/24h)
P=0.027
P=0.364
P=0.229
P=0.574
P=0.776
P<0.001
P=0.053
Baseline
Low Salt
High Salt
SS subjects
SR subjects
b
1000
500
0
-500
-1000
-1500
Urinary UA excretions (μmol/24h)
Low-salt response
High-salt response
P = 0.67
P = 0.386
SS subjects
SR subjects
c
r=--0.075, P=0.483
30
20
10
0
-10
Degreeof Salt senstivity (mmHg)
-3000
-2000
-1000
0
1000
2000
3000
Urinary UA excretion difference (μmol/24h)

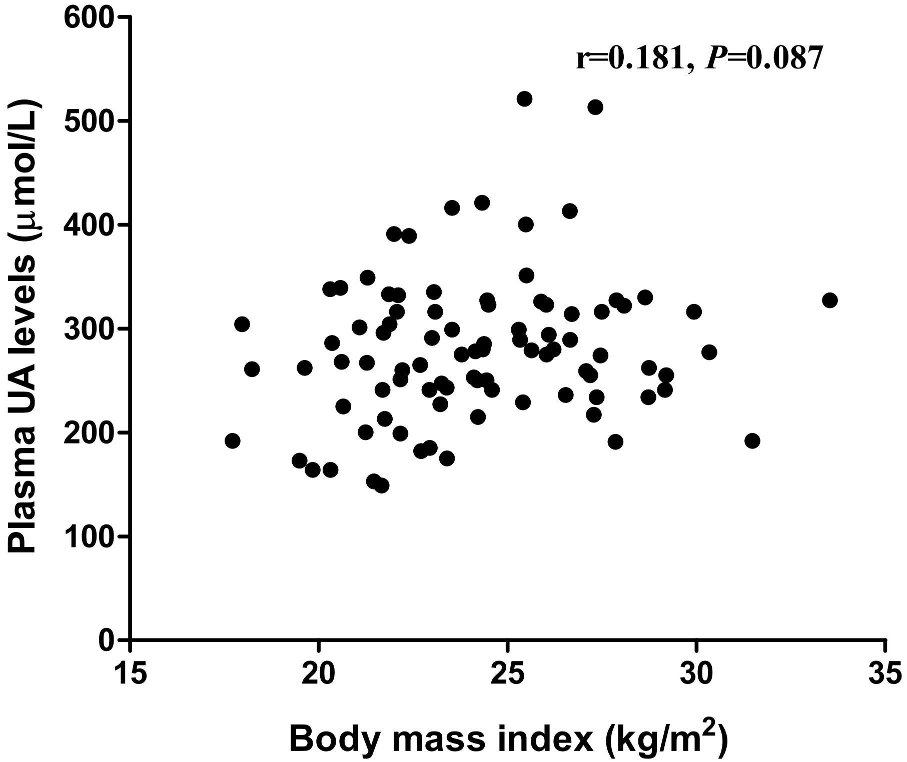

r=0.181, P=0.087
Plasma UA levels (μmol/L)
Body mass index (kg/m2)
0
100
200
300
400
500
600
15
20
25
30
35

Figure Legends

Fig. S1. (a) Comparison of urinary UA excretions at baseline, low-salt and high-salt intake periods in salt-sensitive (SS) and salt-resistant (SR) subjects. Vertical dashed lines represent differences between SS and SR subjects at each of the salt intake levels. Horizontal dashed lines represent differences between salt intake levels (baseline versus low and low versus high). (b) Comparison of urine UA responses to low-salt and high-salt diets in SS and SR subjects. (c) Correlation between urinary UA excretion difference and degree of salt sensitivity in all subjects.

Fig. S2. Correlation between plasma UA levels and body mass index in all subjects.
